# Correlation between heart rate variability and polysomnography-derived scores of obstructive sleep apnea

**DOI:** 10.3389/fnetp.2022.958550

**Published:** 2022-09-06

**Authors:** Rafael Rodrigues dos Santos, Thais Marques da Silva, Luiz Eduardo Virgilio Silva, Alan Luiz Eckeli, Helio Cesar Salgado, Rubens Fazan

**Affiliations:** ^1^ Department of Physiology, School of Medicine of Ribeirão Preto, University of São Paulo, Ribeirão Preto, Brazil; ^2^ Department of Neuroscience and Sciences of Behavior, Division of Neurology, School of Medicine of Ribeirão Preto, University of São Paulo, Ribeirão Preto, Brazil

**Keywords:** sleep, obstructive sleep apnea, polysomnography, heart rate variability, sympathetic

## Abstract

Obstructive sleep apnea (OSA) is one of the most common sleep disorders and affects nearly a billion people worldwide. Furthermore, it is estimated that many patients with OSA are underdiagnosed, which contributes to the development of comorbidities, such as cardiac autonomic imbalance, leading to high cardiac risk. Heart rate variability (HRV) is a non-invasive, widely used approach to evaluating neural control of the heart. This study evaluates the relationship between HRV indices and the presence and severity of OSA. We hypothesize that HRV, especially the nonlinear methods, can serve as an easy-to-collect marker for OSA early risk stratification. Polysomnography (PSG) exams of 157 patients were classified into four groups: OSA-free (*N* = 26), OSA-mild (*N* = 39), OSA-moderate (*N* = 37), and OSA-severe (*N* = 55). The electrocardiogram was extracted from the PSG recordings, and a 15-min beat-by-beat series of RR intervals were generated every hour during the first 6 h of sleep. Linear and nonlinear HRV approaches were employed to calculate 32 indices of HRV. Specifically, time- and frequency-domain, symbolic analysis, entropy measures, heart rate fragmentation, acceleration and deceleration capacities, asymmetry measures, and fractal analysis. Results with indices of sympathovagal balance provided support to reinforce previous knowledge that patients with OSA have sympathetic overactivity. Nonlinear indices showed that HRV dynamics of patients with OSA display a loss of physiologic complexity that could contribute to their higher risk of development of cardiovascular disease. Moreover, many HRV indices were found to be linked with clinical scores of PSG. Therefore, a complete set of HRV indices, especially the ones obtained by the nonlinear approaches, can bring valuable information about the presence and severity of OSA, suggesting that HRV can be helpful for in a quick diagnosis of OSA, and supporting early interventions that could potentially reduce the development of comorbidities.

## Introduction

Obstructive sleep apnea (OSA) is a common respiratory sleep disorder characterized by partial or total airway obstruction episodes, impairing the efficient gas exchange during sleep (Dempsey et al., 2010). It has a high prevalence, affecting almost one billion people worldwide, at least in its mild form of severity (Benjafield et al., 2019). It is well recognized that OSA deteriorates daytime life, increases the incidence of work and traffic accidents (Sassani et al., 2004; Monahan and Redline, 2011), and also leads to the development of comorbidities such as cardiovascular diseases, increasing the risk of life-threatening events (Dempsey et al., 2010).

The hypoxic/hypercapnic episodes suffered by patients with OSA elicit responses, such as sympathetic activation, aiming to re-establish the expected blood oxygen levels. Chronically, recurrent episodes of chemoreceptors activation due to hypoxic events can trigger sustained cardiac dysautonomia, with sympathetic predominance, that can contribute to the development of cardiovascular diseases and increase the risk of cardiac events in patients with OSA (Somers et al., 1995; Somers et al., 2008).

Furthermore, polysomnography (PSG), the gold standard test for the identification and classification of OSA, is a complex and expensive procedure, leading patients to wait months to years before receiving an adequate diagnosis (Tobaldini et al., 2013). It is estimated that about 70% of individuals affected by OSA remain untreated (Sassani et al., 2004; Monahan and Redline, 2011) and are therefore susceptible to the development of severe comorbidities.

The analysis of heart rate variability (HRV) is a valuable probe to investigate the neural control of the heart and is associated with cardiac risk in several diseases, including OSA (Tobaldini et al., 2013; Sequeira et al., 2019; Dissanayake et al., 2021). HRV represents a myriad of indices that describe the dynamics of cardiac intervals on a beat-by-beat basis, usually derived from the electrocardiogram (ECG). Indices of HRV can be obtained using linear or nonlinear approaches (Shaffer and Ginsberg, 2017). The linear approaches are divided into the time and frequency domains. Indices in the time domain provide statistical and geometrical metrics of the cardiac interval. On the other hand, frequency-domain methods, in which spectral analysis is the principal representative, analyze oscillatory, frequency-dependent components of cardiac intervals. In contrast, nonlinear methods of HRV analysis can provide diverse information on cardiac dynamics, which is highly relevant to characterizing the complexity of living organisms (Shaffer and Ginsberg, 2017). Nowadays, more and more nonlinear indices are being proposed, revealing information that linear approaches cannot provide.

Changes in HRV are observed in various diseases, including OSA, since it is well demonstrated that OSA markedly affects the autonomic function, especially in its more severe forms (Tobaldini et al., 2013; Sequeira et al., 2019; Dissanayake et al., 2021). Nevertheless, despite several studies evaluating HRV in patients with OSA, they only assessed a reduced number of HRV indices, mainly focused on linear approaches (Sequeira et al., 2019).

The present study aims to analyze and correlate a variety of HRV indices, calculated with both linear and nonlinear approaches, in patients with different degrees of severity of OSA (mild, moderate, or severe) evaluated by PSG. We hypothesize that the more recent nonlinear methods for assessing HRV, included in this study, provide important markers for stratifying OSA severity.

## Methods

### Patients

PSG exams were performed at the University Hospital of Ribeirão Preto Medical School of the University of São Paulo (HC-FMRP/USP) between 2015 and 2021. The data collection and the analysis protocols were carried out in accordance with The Code of Ethics of the World Medical Association (Declaration of Helsinki) and authorized by the Research Ethics Committee of HC-FMRP/USP (Protocol # 42058720.6.000.5440/4.550.2327).

Patients older than 18 years old, with a minimum sleep recording period of 5 h and 15 min in the PSG, and with RR series containing no more than 2.5% of artifacts identified (see next section) were included in the study. Out of 301 exams collected, 144 were not used due to problems with the recording files, such as insufficient collected time, corrupted files, poor signal quality, and/or arrhythmias (96 recordings); exams from patients under 18 years old (2 recordings); and exams with missing data in the report (46 recordings). The final sample was composed by 157 recordings.

### Data processing

The PSG exam records variables such as electroencephalogram (EEG), electrooculogram (EOG), electromyogram (EMG), electrocardiogram (ECG), thoracic and abdominal movements by piezo-electric straps, pulse oximetry, nasal pressure transducer system and nasal and mouth thermocouple airflow sensor to monitor the airflow, microphone to detect snores, sensor to determine body position, and a video camera to monitor the patient during sleep. So, ECG recordings from the PSG exams (sampling rate: 512 Hz) were obtained and, after visual inspection, segments of 15 min from each hour were extracted for each patient from the first 6 h of recording. The segment selection was based on a visual assessment on the quality of the ECG, avoiding the presence of artifacts as much as possible. Time series of successive RR intervals were generated (ECG Module for LabChart, AD Instruments, Dunedin, New Zealand) for each 15-min segment and corrected for remaining artifacts and/or ectopic beats. Artifacts were identified as follows: first, a moving median window of size *W* was applied to the RR series, creating a median line. Next, a lower and upper tolerance were defined as the median line shifted down and up, respectively, by a factor of *T* (tolerance). This tolerance corresponds to a percentage of the average median line. The optimal values of *W* and *T* were manually chosen for each series, varying in the range *W* = [5, 70] and *T* = [0.01, 0.80]. Finally, all RR values below the lower or above upper tolerance were replaced using linear interpolation. When the number of corrections exceeded 2.5% of the total number of beats, the patient was excluded from the study (10 RR series were excluded from the study for this reason).

### HRV analysis

Corrected RR series were used to calculate HRV indices using the computer software PyBios (Silva et al., 2020). Linear indices were calculated in time and frequency domains. In the time-domain, we calculated the standard deviation of RR values (SDNN) and root mean square of successive RR differences (RMSSD). For frequency-domain analysis, RR series were resampled at 3 Hz by cubic spline interpolation and divided into segments of 512 values overlapped by 50%. Following, after the application of a Hanning window, the segments had their spectra calculated by the periodogram (Fourier transform) and were integrated into bands of low- (LF: 0.04–0.15 Hz) and high-frequency (HF: 0.15–0.4 Hz). The power of the spectra in the LF band was assessed in normalized units (LFnu), while HF power was evaluated in absolute units (HFabs). The LF/HF ratio (both in absolute units) was also calculated (Montano et al., 1994; Silva et al., 2017).

Several nonlinear indices of HRV were also calculated. The evaluation of fractal behavior (self-similarity) of RR intervals series was performed by the detrending fluctuation analysis (DFA) in the scaling range 5 < *n* < 15 (α1), where *n* is the number of RR intervals considered (Peng et al., 1995). Entropy measures assess the irregularity (unpredictability) of RR patterns. Here, we calculated the following entropies: sample entropy (SampEn; sequence length *m* = 2; tolerance *r* = 0.15), fuzzy entropy (FuzzyEn; sequence length *m* = 2; tolerance *r* = 0.15; fuzzy exponent *n* = 2), distribution entropy (DistEn; sequence length *m* = 3; number of bins *M* = 512), dispersion entropy (DispEn; sequence length *m* = 3; number of classes *nc* = 6), permutation entropy (PermEn; sequence length *m* = 3; noise added to deal with equal values), attention entropy (AttEn), and phase entropy (PhaseEn; number of sectors *k* = 16). The details of the calculation of these approaches are described elsewhere (Richman and Moorman, 2000; Chen et al., 2007; Li et al., 2015; Rostaghi and Azami, 2016; Ribeiro et al., 2021). The acceleration-deceleration capacity (AC/DC) was also calculated. It considers the average magnitude of increases and decreases in heart rate regarding their adjacent values (Bauer et al., 2006). Besides, three asymmetry indices were used to evaluate whether the changes in RR intervals are similar when the series is time-reversed: Porta’s, Guzik’s, and Ehlers’ indices (Guzik et al., 2006; Porta et al., 2008). For Porta’s and Guzik’s indices, symmetric RR series are characterized by values near 50, representing the balance between positive and negative variations within the series. In contrast, Ehler’s index is based on the skewness of RR differences, and values near zero represent symmetric (time-reversible) series. In addition, two symbolic dynamics analyses were calculated. The method proposed by Porta and co-workers was calculated as described elsewhere (Porta et al., 2007a). Briefly, this method divides the overall range (max-min) of RR values into six equally distributed bins and symbolizes the values according to the level it belongs. Following, it does compute the percentage of patterns assigned as 0V (zero variation), 1V (one variation), 2LV (two like variations), and 2UV (two unlike variations), where sequences of three consecutive symbols represent the patterns. Another symbolic dynamics method calculated was the binary symbolic dynamics, as described by Cysarz et al. (2015). Differently from Porta’s approach, the binary method considers as patterns the sequences of accelerations and decelerations of heart rate. Only patterns of type 0, 1, and 2V (two variations) are possible in this case, creating the Bin-0V, Bin-1V, and Bin-2V indices, respectively. For both approaches of symbolic analysis, 0V represents the percentage of patterns with the slowest oscillations, while 2UV (or 2V) represents the percentage of patterns with the fastest oscillations. Finally, we also evaluated some heart rate fragmentation (HRF) indices. HRF was proposed by Costa et al. (2017a); Costa et al. (2017b) and is intended to quantify ultra-fast (erratic) variations in heart rate. Here, we used the symbolic dynamics approach of HRF, although it is essentially different from the two previously described symbolic dynamics approach. In HRF, sequences of four consecutive RR differences are classified as words containing zero (W0), one (W1), two (W2), or three (W3) inflection points. The general percentage of inflection points (PIP) was also calculated.

### Classification of OSA

The PSG report, provided by a qualified physician, was used as the source of indices of interest to determine the presence and severity of OSA, which were: apnea/hypopnea index (AHI), arousal index (AI), percentage of the total sleep time that the patient achieved oxygen saturation below 90% (T90), and oxygen saturation nadir during sleep (SatMin). The AHI represents the number of apneic/hypopnea events divided by the total hours of sleep. An apnea event is defined as a reduction >90% of the airflow, lasting at least 10 s (associated with a respiratory effort), and the hypopnea event is characterized by a reduction >30% of the airflow in a period >10 s, associated with an oxygen desaturation >3% during this period or with arousal (Iber et al., 2007). AHI is the most used reference to diagnose OSA and establish its severity class. Here, we defined four groups of patients according to their AHI: if below 5, the patient is considered normal (OSA-free); when it is between 5 and 15, 15 and 30, and higher than 30, OSA is diagnosed and classified as mild, moderate or severe, respectively (Sleep-related breathing disorders in adults, 1999). The AI represents the number of arousals divided by total sleep time. Arousal is characterized by an abrupt shift (a return to alpha or theta waves) seen in the electroencephalogram (EEG) that lasts at least 3 s, with at least 10 s of stable sleep preceding the change, and additionally, an alteration in electromyogram (EMG) during REM sleep (Martin et al., 1997; Iber et al., 2007; Taylor et al., 2016).

### Statistical analysis

The Shapiro-Wilk test was used to check the normality of each variable. Since most variables showed a non-normal distribution, the results are shown as median (first and third quartiles), and the Kruskal-Wallis test was applied to compare groups. When differences were observed, the *post-hoc* test of Dunn was applied. For the gender differences between groups, the chi-square test was applied. Finally, the Spearman’s correlation coefficient was used to evaluate the correlation strength between HRV and PSG indices. In all cases, statistical significance was considered when *p* ≤ 0.05.

## Results

**FIGURE 1:**
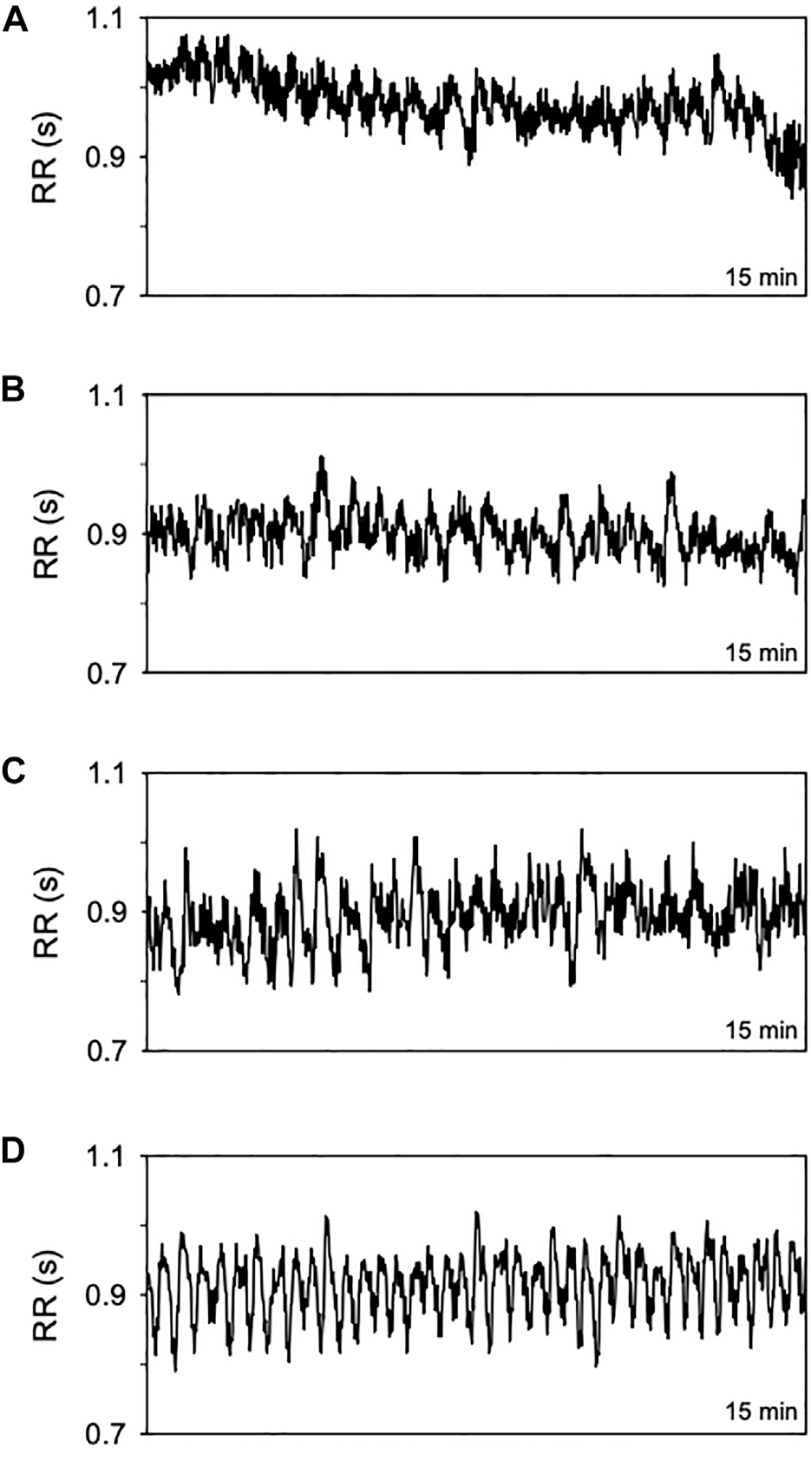
Representative time series of RR intervals (15 min) from a normal subject **(A)** and patients with OSA in its mild **(B)** moderate **(C)**, and severe form **(D)**.

Figure 1 shows series of RR intervals from one patient of each group evaluated. The number of patients in each group studied ranged from 24 to 57. Normal subjects (OSA-free) formed the smaller group, while the larger one encompasses patients with severe OSA. AI, T90, and SatMin were not different in the mild form of OSA compared to normal subjects. Nevertheless, except for the SatMin, all PSG indices were higher in patients with moderate and severe OSA forms than normal individuals (Table 1).

**TABLE 1:** PSG indices in normal subjects and patients with OSA under three severity classes.

	Normal (*n* = 24)	Mild (*n* = 39)	Moderate (*n* = 37)	Severe (*n* = 57)
Males	5 (20.8%)	19 (48.7%)	17 (45.9%)	26 (45.6%)
Age	36 (28–54)	50 (39–63)	58 (47–66)*	55 (40–63)*
BMI	27.6 (25.2–31.8)	31.4 (27.7–33.71)	30.0 (27.4–3 7.3)	34.5 (29.0–38.6)*
AHI	2.8 (1.1–3.5)	10 (8–13)*	23 (19–26)*^#^	56 (39–79)*^#$^
AI	21 (12–32)	31 (17–38)	35 (24–56)*	60 (43–80)*^#$^
T90	0.05 (0–0.47)	0.8 (0.1–8.1)	2.2 (0.3–10)*	12 (4–39)*^#$^
SatMin	88 (84–91)	86 (81–89)	84 (77–87)*	75 (65–82)*^#$^

BMI, body mass index; AHI, apnea/hypopnea index; AI, arousal index; T90, percentage of total sleep time of oxygen saturation below 90%; SatMin, oxygen saturation nadir (%) during sleep; Values are presented as Median (first quartile, third quartile); *p < 0.05 versus normal; ^#^ *p* < 0.05 versus mild; ^$^ *p* < 0.05 versus moderate.

Table 2 presents the mean RR interval and HRV indices calculated in all groups of patients evaluated. Surprisingly, no differences were found in either mean RR interval or HRV indices calculated in the time domain among the healthy individuals and patients with OSA under any class of severity. On the other hand, the spectral analysis showed results compatible with the cardiac autonomic imbalance, with sympathetic predominance, in patients with moderate and severe forms of OSA (increase in LFnu and decrease in HFabs in moderate OSA as compared to normal subjects, and a monotonic increase in LF/HF with OSA severity). Similarly, the symbolic dynamics revealed an increase in the occurrence of 0V and a decrease in 2LV in patients with moderate and severe forms of OSA, as compared to normal controls. For the binary symbolic dynamics method, 1V was lower in all classes of OSA when compared with normal subjects.

**TABLE 2:** Cardiac interval and HRV indices calculated from normal individuals and patients with OSA at distinct classes of severity.

	Normal (*n* = 24)	Mild (*n* = 39)	Mod. (*n* = 37)	Severe (*n* = 57)
RR (ms)	**875** (796–936)	**886** (803–1,063)	**905** (816–1,024)	**876** (801–963)
SDNN (ms)	**43** (29–64)	**34** (28–44)	**36** (31–51)	**44** (34–68)
RMSSD (ms)	**31** (17–60)	**22** (12–34)	**18** (13–30)	**26** (17–36)
LF power (nu)	**41** (29–52)	**48** (31–65)	**54** (40–70)*	**51** (35–65)
HF power (ms^2^)	**352** (127–1,183)	**150** (57–344)	**133** (54–326)*	**285** (101–501)
LF/HF	**1.1** (0.5–1.7)	**1.4** (0.9–2.5)	**1.8** (1.1–5.3)*	**1.9** (0.7–3.4)*
0V (%)	**26** (10–31)	**31** (20–42)	**35** (25–46)*	**36** (22–44)*
1V (%)	**47** (42–50)	**45** (40–48)	**44** (39–47)	**44** (40–49)
2LV (%)	**9.3** (5.3–16.8)	**6.8** (4.2–9.5)	**4.3** (2.9–7.8)*	**5.5** (2.6–11.8)*
2UV (%)	**18** (13–24)	**16** (10–25)	**14** (7–21)	**12** (9–18)
DFA-α1	**0.98** (0.84–1.17)	**1.10** (0.93–1.34)	**1.20** (1.08–1.43)*	**1.27** (0.92–1.41)*
SampEn	**1.84** (1.75–1.95)	**1.81** (1.61–1.95)	**1.79** (1.35–2.02)	**1.66** (1.49–1.88)*
FuzzyEn	**1.62** (1.53–1.88)	**1.52** (1.33–1.75)	**1.51** (1.16–1.64)*	**1.37** (1.23–1.66)*
DistEn	**0.64** (0.56–0.73)	**0.58** (0.54–0.67)	**0.59** (0.55–0.66)	**0.62** (0.56–0.69)
PermEn	**2.46** (2.43–2.51)	**2.48** (2.43–2.53)	**2.48** (2.41–2.55)	**2.47** (2.43–2.52)
DispEn	**4.46** (4.35–4.71)	**4.35** (4.07–4.66)	**4.28** (3.83–4.52)*	**4.16** (3.96–4.47)*
AttEn	**1.92** (1.56–2.06)	**1.94** (1.65–2.30)	**2.03** (1.69–2.60)	**2.25** (1.87–2.44)*
PhaseEn	**0.92** (0.91–0.93)	**0.91** (0.89–0.92)	**0.91** (0.89–0.92)	**0.92** (0.91–0.93)
AC (ms)	−**12.3** (−17.9 to −7.7)	−**9.0** (−11.5 to −5.9)	−**8.2** (−11.3 to −5.5)*	−**11.4** (−15.9 to −6.8)
DC (ms)	**11.5** (7.3–19.2)	**8.8** (5.8–11.2)	**8.5** (5.6–11.6)	**11.2** (7.2–15.9)
Porta (%)	**49.4** (48.1–52.0)	**50.0** (48.3–51.3)	**50.4** (49.4–51.7)	**50.1** (48.4–52.3)
Guzik (%)	**49.6** (48.9–53.5)	**50.8** (48.6–52.3)	**51.5** (49.7–52.6)	**50.6** (48.5–52.8)
Ehlers	**0.06** (-0.14–0.83)	**0.11** (−0.12 to 0.69)	**0.39** (−0.04 to 0.59)	**0.18** (−0.24 to 0.70)
Bin-0V (%)	**17.4** (11.7–21.1)	**16.4** (11.0–25.4)	**17.9** (10.6–31.7)	**22.5** (15.5–27.1)
Bin-1V (%)	**64.8** (62.1–73.1)	**61.5** (55.4–67.6)*	**61.3** (50.1–64.0)*	**58.0** (52.9–64.8)*
Bin-2V (%)	**16.0** (10.3–19.7)	**19.6** (13.0–28.4)	**18.9** (13.5–23.0)	**17.3** (14.0–23.0)
PIP (%)	**53.2** (49.3–58.5)	**58.3** (52.8–62.2)	**55.5** (51.5–61.0)	**54.7** (50.2–60.3)
W0 (%)	**3.16** (1.62–4.49)	**3.84** (1.94–6.02)	**4.29** (2.14–10.75)	**6.20** (2.99–9.12)* ^**#**^
W1 (%)	**42.5** (30.8–51.0)	**30.7** (21.0–41.7)*	**33.4** (23.4–42.6)*	**35.2** (26.6–44.5)
W2 (%)	**42.2** (39.1–52.0)	**47.0** (41.8–59.2)	**46.7** (38.1–54.1)	**42.9** (35.3–50.2)
W3 (%)	**10.4** (5.3–13.3)	**12.6** (9.9–19.7)	**12.1** (9.2–21.1)	**13.1** (7.8–17.9)

RR, mean RR interval; SDNN, standard deviation of normal-to-normal cardiac intervals; RMSSD, root mean square of successive RR differences; LF, low-frequency band; HF, high-frequency band; LF/HF: the ratio between the powers at LF and HF bands; 0V, percentage of patterns with zero variations; 1V, percentage of patterns with one variation; 2LV, percentage of patterns with two like variations; 2UV, rate of patterns with two unlike variations; SampEn, sample entropy; DFA-α1, detrended fluctuation analysis (5 ≤ *n* ≤ 15); FuzzyEn, fuzzy entropy; DistEn, distribution entropy; PermEn, permutation entropy; DispEn, dispersion entropy; AttEn, attention entropy; PhaseEn, phase entropy; AC, acceleration capacity; DC, deceleration capacity; Porta, Porta’s asymmetry index; Guzik, Guzik’s asymmetry index; Ehlers, Ehlers’ asymmetry index; Bin-0V, patterns with 0 variation (binary method); Bin-1V, patterns with one variation (binary method); Bin-2V, patterns with two variations (binary method); PIP, percentage of inflection points; W_0_, percentage of patterns with zero inflection points; W_1_, percentage of patterns with one inflection point; W_2_, percentage of patterns with two inflection points; W_3_, percentage of patterns with three inflection points; Values in bold are presented as median (1° quartile, 3° quartile); *p < 0.05 versus normal; ^#^ *p* < 0.05 versus mild.

Regarding the entropy measures, SampEn, FuzzyEn, and DispEn showed a reduction in patients with moderate or severe forms of OSA, while AttEn increased in severe patients compared to normal individuals. DistEn, PermEn, PhaseEn were not significantly different among groups. The DFA short-term scaling exponent was found higher in patients with moderate and severe OSA as compared to healthy individuals. The AC/DC method showed that only the acceleration capacity is reduced in a moderate form of OSA. HRF showed that the occurrence of W0 patterns is increased in the severe OSA as compared to mild OSA and normal subjects. The rate of occurrence of W1 patterns is decreased in mild and moderate OSA compared to the normal group. Moreover, similar to the LF/HF ratio, 0V from symbolic analysis, DFA-α1, AttEn, the W0 index of HRF showed a linear increase as the severity of OSA rises. None of the three asymmetry indices are different among all groups.

**FIGURE 2:**
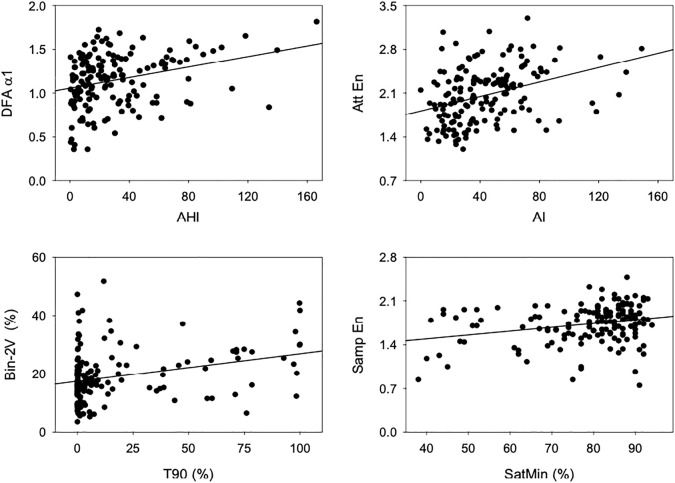
Scatterplots and linear regression lines, showing the relationship between indices of HRV and PSG. Top left: DFA-α1 and AHI, top right: AttEn and AI, bottom left: Bin-2V and T90, and bottom right: SampEn and SatMin.

Figure 2 shows scatter plots and regression lines of HRV indices that showed high correlation with PSG-derived scores. Table 3 shows Spearman’s correlation coefficient among indices of HRV and indices of OSA, the latter obtained from the reports of PSG exams. The mean RR interval, the HRV measured in the time domain, and all the asymmetry indices showed no significant correlation with PSG-derived indices of OSA. The power of RR spectra at LF band, calculated by spectral analysis, showed a significant positive correlation with both AHI and AI. AHI and AI were found to be positively correlated with the occurrence of 0V, and negatively correlated with 2LV and 2UV from the symbolic analysis. The percentage of 2LV patterns was also negatively correlated with T90. A similar result was observed for the binary symbolic dynamics, i.e., Bin-0V was positively correlated with AHI and AI, while Bin-1V index was negatively correlated with them. On the other hand, Bin-2V showed a positive correlation with T90 and a negative correlation with the SatMin. Bin-1V was negatively correlated with T90. The acceleration and deceleration capacities were positively and negatively associated with T90, respectively. From HRF indices, the PIP showed a positive correlation with T90, while W0 was found positively correlated with AHI and AI. W1 and W2 indices showed a negative correlation with T90 and AI, respectively. Finally, the W3 showed a positive correlation with AHI and T90. From the set of entropy methods, SampEn, FuzzyEn, and DispEn were correlated to all the four PSG-derived OSA indices. The same occurred for DFA-α1 and LF/HF ratio. In all situations, the correlation direction was the same for AHI, AI, and T90, but the opposite for SatMin. Of note, DFA-α1, FuzzyEn, DispEn, and W0 were the HRV indices that displayed the strongest correlations with AHI (0.30), while AttEn showed the highest overall correlation coefficient obtained with AI (0.40).

**TABLE 3:** Correlation coefficients between HRV and PSG indices.

	AHI	AI	T90	SatMin
Mean RR	−0.04	−0.13	−0.10	0.13
SDNN	0.13	0.11	−0.08	−0.05
RMSSD	−0.04	−0.09	−0.10	0.01
LF power (nu)	0.21*	0.32*	0.13	−0.12
HF power (ms^2^)	−0.05	−0.09	−0.15	0.03
LF/HF	0.24*	0.31*	0.16*	−0.17*
0V	0.26*	0.30*	0.13	−0.10
1V	−0.11	−0.02	−0.06	−0.01
2LV	−0.24*	−0.22*	−0.20*	0.12
2UV	−0.23*	−0.35*	0.01	0.03
DFA-α1	0.30*	0.32*	0.19*	−0.19*
SampEn	−0.29*	−0.31*	−0.23*	0.24*
FuzzyEn	−0.30*	−0.34*	−0.20*	0.18*
DistEn	−0.01	0.00	−0.05	−0.15
PermEn	−0.01	−0.19*	0.18*	−0.06
DispEn	−0.30*	−0.34*	−0.18*	0.17*
AttEn	0.29*	0.40*	0.05	−0.06
PhaseEn	0.08	0.17*	−0.11	−0.01
AC	0.01	0.00	0.17*	−0.04
DC	0.00	0.01	−0.17*	0.03
Porta	0.06	0.01	0.00	0.00
Guzik	0.06	0.00	−0.02	0.00
Ehlers	0.04	−0.02	−0.04	0.01
Bin-0V	0.23*	0.38*	−0.01	−0.05
Bin-1V	−0.28*	−0.25*	−0.23*	0.16
Bin-2V	0.10	−0.04	0.28*	−0.17*
PIP	0.01	−0.14	0.23*	−0.05
W0	0.30*	0.39*	0.05	−0.14
W1	−0.08	0.05	−0.26*	0.11
W2	−0.11	−0.23*	0.06	0.03
W3	0.17*	0.08	0.25*	−0.09

Numbers in bold with an asterisk: significant relationship (*p* < 0.05).

## Discussion

The present study evaluated the relationship between HRV indices, calculated by several approaches, and four important PSG-derived clinical scores of OSA. Studies using ECG-based methods as a screening tool for patients with OSA have been described before (Guilleminault et al., 1984). However, to the best of our knowledge, this is the first study to evaluate the relationship of OSA, from its mild to severe forms, with a large set of HRV indices, especially those calculated from nonlinear approaches.

### HRV analysis between normal and OSA individuals

The HRV indices calculated in the time domain were extensively demonstrated to be associated with cardiac risk in several situations (Heart rate variability, 1996). To our surprise, those indices, as well as the mean RR interval, were found to be similar among normal subjects and patients with OSA, even under its more severe form. Nevertheless, conflicting findings of time-domain indices, especially for SDNN, have been reported in patients with OSA, drawing attention to the necessity for more robust HRV approaches to characterize these patients (Roche et al., 1999; Porta et al., 2007b; Cysarz et al., 2013; Kim et al., 2015; Aeschbacher et al., 2016; Nastałek et al., 2019; Spellenberg et al., 2020).

Our findings from spectral analysis and symbolic dynamics strongly suggest a cardiac autonomic imbalance, with sympathetic predominance, in patients with OSA. It is well accepted that high values of the LF/HF ratio and 0V, together with the reduction of 2UV symbols, are linked to an increase in sympathetic and a decrease in parasympathetic cardiac modulation, respectively (Porta et al., 2007b). Additionally, the binary symbolic analysis showed that the occurrence of 1V patterns is reduced in all classes of OSA as compared to healthy individuals. Studies by Cysarz et al. (2013) and Spellenberg et al. (2020) discuss a possible interpretation of the 1V of the binary method as a marker of parasympathetic modulation since it is reduced with stressor tests. Therefore, our findings corroborate several studies by showing that OSA patients tend to have a sympathovagal balance shifted to the sympathetic predominance as compared to healthy subjects. Importantly, studies showed that this sympathetic overactivation extends to the wake period, leading, therefore, to a higher risk of developing cardiovascular diseases (Somers et al., 1995; Porta et al., 2007b; Somers et al., 2008; Shaffer and Ginsberg, 2017). It is believed that this autonomic imbalance might be caused by the intermittent hypoxia that these patients suffer during the night due to recurrent apneic events. The physiopathology of sustained sympathetic overactivity may undoubtedly be related to the frequent activation of chemoreceptors in the carotid body, activating the sympatho-excitatory neural pathways, trying to maintain blood gases homeostasis (Gharibeh and Mehra, 2010).

From nonlinear methods, fractal and entropy measurements were found altered in patients with OSA, which is in line with several previous observations in the literature (Penzel et al., 2003a; Al-Angari and Sahakian, 2007; Sequeira et al., 2019). The DFA short-term fractal exponent (α1) used in this study describes short-term fluctuations at different time scales (Shaffer and Ginsberg, 2017), and was demonstrated to be a valuable parameter for distinguishing the severity of OSA. Apropos, Penzel et al. (2003b) showed that DFA-α1 was a better predictive tool in OSA than the spectral analysis indices. However, opposite findings were also reported, such as those from Silva and co-workers (da Silva et al., 2015), showing that both frequency domain indices and DFA-α1 do not differ between OSA classes and are not significantly correlated with PSG scores. From entropy measurements, results with SampEn, FuzzyEn, and DispEn showed a very similar profile, decreasing with the rise of OSA severity. In contrast, AttEn tends to increase with OSA severity, whereas DistEn, PermEn, and PhaseEn did not differ concerning the severity of the groups. Although AttEn is a very recent and intricate method whose interpretation is still to be better elucidated, SampEn, FuzzyEn, and DispEn are widely recognized as irregularity or unpredictability measurements. In this case, the higher the entropy, the higher the unpredictability of the series. Al-Angari and Sahakian (2007) study also found a significant reduction in SampEn in patients with OSA, supporting the notion that these patients have a more predictable heart rate oscillation. Altogether, those findings with DFA and entropy indicate that patients with OSA have altered fractal dynamics and decreased unpredictability of heart rate oscillations, a condition consistent with loss of physiological complexity in patients with OSA (Goldberger et al., 2002; Burggren and Monticino, 2005; Porta et al., 2007a; Arsac and Deschodt-Arsac, 2018).

Although none of the asymmetry indices were found different among groups, the acceleration capacity is decreased in patients with moderate OSA. Therefore, OSA seems not to affect the number of accelerations and decelerations but may affect the magnitude of heart rate accelerations. In agreement with this idea, the study from Guzik et al. (2013) showed that patients with severe OSA had a reduction in short acceleration and deceleration runs and an increase in long acceleration runs compared with other OSA classes or healthy individuals. Similarly, a study from Jiang et al. (2017) showed that OSA reduces the short acceleration runs and increases both acceleration and deceleration of long runs. Thus, the magnitude of accelerations and decelerations seems to be an important marker to be evaluated in patients with OSA under different forms of severity.

Another promising nonlinear approach to evaluating HRV is heart rate fragmentation. We showed that the occurrence of W0 patterns is increased in severe OSA, while a reduction in W1 patterns occurred in mild and moderate forms of OSA, when compared to healthy subjects. In the first studies conducted by Costa et al. (2017a), Costa et al. (2017b), fragmented indices (such as PIP and W3) were demonstrated to be higher in patients with high cardiovascular risk, whereas a reduced value of fluent indices (such as W0 and W1) were observed in these patients (Costa et al., 2017a; Costa et al., 2017b). However, the same authors draw attention to the fact that W0 patterns should be cautiously interpreted in special populations, such as patients with OSA. An excessive percentage of W0 can be due to an abnormal increase in long acceleration/deceleration runs, which would not be related to a better prognosis. This hypothesis may explain our findings with W0, which also agrees with the observations that OSA increases the acceleration and deceleration of long runs (Guzik et al., 2013; Jiang et al., 2017). Nevertheless, compared to the control group, the lower occurrence of W1 observed in patients with OSA suggests that OSA diminishes the presence of fluent patterns, even though the fragmented patterns (PIP and W3) did not increase.

### Correlation between HRV and PSG indices

The correlation analysis showed that many HRV indices are slightly but significantly correlated with PSG scores. In general, AI showed higher correlation coefficients with HRV than those with AHI, T90, or SatMin. This suggests that the autonomic imbalance of OSA is more related to sleep fragmentation than to the number of apneic/hypopneic events. This is in agreement with several studies that evaluated the association of HRV with several sleep disorders (Morrell et al., 2000; Sforza et al., 2007; Taylor et al., 2016). Here, time-domain and asymmetry indices did not show any significant correlations with PSG scores, but the LF, LF/HF, and 0V showed a positive correlation with AHI and AI, while 2LV and 2UV showed a negative relationship with these PSG-indices. Previous studies reported similar findings. Kim et al. (2019) showed that AI is an independent factor for an increase in LF power of RR spectra, and both AHI and AI independently contributed to a reduction of HF power and an increase of LF/HF. In another study, Gong et al. (2016) found a significant relationship between frequency domain indices and the AHI and AI. Besides, in a multiple regression analysis, AI had a substantial relationship with LF/HF. In addition, Park et al. (2008) demonstrated that LF/HF showed a good correlation with AHI. All these findings strengthen the notion that apneic events and sleep fragmentation are important contributors to sympathetic overactivity, leading to a higher risk of developing cardiovascular diseases in patients with OSA.

Unlike studies with classical linear HRV approaches, far fewer studies can be found evaluating the relationship between nonlinear HRV and OSA. Here, we demonstrated that DFA (short-term exponent) and some entropies are correlated, although slightly, with the top PSG scores used to detect and classify OSA, i.e., AHI, AI, T90, and SatMin. While SampEn, FuzzyEn, and DispEn showed an inverse relationship with AHI and MI, DFA-α1 and AttEn showed positive associations. The importance of entropy and fractal dynamics in the prognosis of diseases is widely demonstrated (Voss et al., 2009; Sassi et al., 2015). Here, we showed that those HRV nonlinear indices correlate to all the clinical scores studied, pointing to their superiority over classical HRV indices and their importance for OSA prognostication. The HRF, a recent and promising tool, showed that both indices reflecting fragmented patterns (PIP and W3) positively correlated with T90, while W3 also had a positive correlation with AHI. This direct association between fragmented patterns and OSA severity points to the degradation of heart rate control with OSA. On the other hand, W0, which is usually considered a marker of fluent patterns, reached one of the strongest correlations with both AHI and AI. However, as mentioned before, the increase of W0 with OSA severity is related to the long heart rate acceleration and deceleration runs caused by the disease. Therefore, HRF indices seem to be a valuable source of information to assess the severity of OSA.

The results presented here using a large number of HRV indices advocates for the importance of using a comprehensive set of HRV indices in the characterization of OSA and its severity. Since no single HRV index was highly correlated to OSA severity markers, the different HRV indices may provide complementary information. Studies combining those indices through machine learning models are being conducted to identify the importance of each one when they are combined in a single predictive model.

### Limitations

Finally, it is essential to highlight some limitations of our study. First, the groups are unbalanced regarding the number of patients because patients with severe OSA represent the most prevalent group. Second, the segments of HRV obtained from PSG recordings were not standardized regarding the sleep phase or the presence of apneic/hypopneic episodes. And third, we did not consider comorbidities and medications in use by the patients. Further studies are necessary to evaluate the influence of those factors on the predictive value of HRV metrics in OSA.

## Conclusion

In the present study we evaluated the relationship between a wide variety of HRV indices and the presence/severity of OSA. In addition to changes in several indices of HRV in patients with OSA, we showed a significant relationship in a number of these indices with important PSG scores commonly used to define the presence and severity of this disease. More specifically, we highlight LF/HF ratio, 2LV, DFA-α1, SampEn, FuzzyEn, DispEn, and Bin-1V as the most relevant, since they showed significant correlation to at least three out of four OSA severity indices. Our findings point to HRV indices, especially nonlinear ones, as proper adjuvant markers in OSA stratification, which can help to screen these patients, aiming for a quick diagnosis and prevention of the risk of developing the comorbidities related to this clinical syndrome.

## Data Availability

The raw data supporting the conclusion of this article will be made available by the authors, without undue reservation.
